# Has the Russian invasion of Ukraine reinforced anti-globalization sentiment in Austria?

**DOI:** 10.1007/s10663-023-09572-1

**Published:** 2023-03-15

**Authors:** Jerg Gutmann, Hans Pitlik, Andrea Fronaschütz

**Affiliations:** 1 Institute of Law and Economics, University of Hamburg, Johnsallee 35, 20148 Hamburg, Germany; 2 University of Freiburg, Freiburg i.Br., Germany; 3 CESifo, Munich, Germany; 4 Austrian Institute of Economic Research, Vienna, Austria; 5 Austrian Gallup Institute, Vienna, Austria

**Keywords:** Austria, Crisis, Conflict, Globalization attitudes, Russian invasion, War, F13, F51, F52, N40

## Abstract

The Russian invasion of Ukraine has caused disruptions in international trade and highlighted the dependency of small open economies in Europe on imports, especially of energy. These events may have changed Europeans’ attitude towards globalization. We study two waves of representative population surveys conducted in Austria, one right before the Russian invasion and the other two months later. Our unique dataset allows us to assess changes in the Austrian public’s attitudes towards globalization and import dependency as a short-term reaction to economic turbulences and geopolitical upheaval at the onset of war in Europe. We show that two months after the invasion, anti-globalization sentiment in general has not spread, but that people have become more concerned about strategic external dependencies, especially in energy imports, suggesting that citizens’ attitudes regarding globalization are differentiated.

**Supplementary Information:**

The online version contains supplementary material available at 10.1007/s10663-023-09572-1.

## Introduction

On February 24, 2022, Russia launched a brutal invasion against Ukraine that shocked the civilized world and was condemned by 78% of all states in the UN General Assembly. Following the Great Recession and a still abounding COVID-19 pandemic, the Russia-Ukraine war turns out to be a third major worldwide crisis in less than two decades with potentially significant effects on international trade and commerce. Already before the war, the public in many countries appear to have turned their back on economic globalization. Grassroots movements, mainly from the political left, organized opposition against international trade agreements, causing serious concerns about a return of protectionism (Rodrik 2018). It has been argued (see, e.g., Posen 2022) that the Russian invasion and the resulting sanctions will add to the corrosion of public support for globalization—and eventually of globalization itself—that has so far been driven by the rising influence of populist and nationalist political leaders and the West’s erection of barriers to Chinese economic integration.

Theoretically, the Russian invasion of Ukraine could have two opposing effects on attitudes towards globalization. The experience of economic instability due to close trade ties with Russia in the energy sector and with Ukraine in the food sector could strengthen citizens’ desire for national autarky and, thus, less dependence on international trade. This is explained in Rodrik’s (2021) theoretical framework as either a direct effect of economic conditions on policy preferences or indirectly by changing identity or the salience of cultural values, for example, due to heightened feelings of insecurity. However, the dependence on Russia as a major trade partner for natural resources could also motivate calls for more globalization to diversify risk by establishing trade ties for critical imports with a variety of nations (e.g., Boute 2022). A theoretical prediction which of these effects dominates does not appear feasible, consistent with Acemoglu et al.’s (2009) argument that such critical junctures can lead to divergent political–economic development paths. To test the relative importance of the two effects, we scrutinize how anti-globalization sentiment in Austria has changed in the two months after the invasion.

Austria is an interesting case study, as it is highly dependent on gas imports from Russia and its population is generally skeptical of globalization. First, Austria has a history of anti-free trade activism that has, for example, produced strong political opposition against TTIP in 2014 (Pitlik 2016). Nationalist and anti-globalization sentiments are also reflected in the electoral success of a nationalist and populist party.^1^ Second, Austria is highly dependent on Russian energy imports (in 2021, almost 90% of its gas was supplied by Russia). According to a May 2022 Special Business Survey on the Russia-Ukraine war (Hölzl et al. 2022), 68% of manufacturers in Austria report production impediments. Rising energy and food prices also affect private households and have incited voluminous fiscal support programs by the Austrian government. Given Austria’s economic exposure and its citizens’ generally critical attitudes towards globalization, an anti-globalization effect from Russia’s invasion on the Austrian population could be interpreted as an upper bound for a plausible effect of the invasion on attitudes in most other European countries.

We make use of two waves of the Gallup Austria population survey—one conducted immediately before Russia’s invasion of large parts of Ukraine in February 2022 and the other two months later—to examine the immediate impact of the first months of the Russia-Ukraine war on public attitudes towards globalization in general and towards import dependency, particularly regarding energy. A period of two months for our treatment to take effect is sensible for several reasons. First, Dräger et al. (2022) show that it takes time for the general population to feel and understand the economic consequences of the invasion; and Ropele and Tagliabracci (2022) demonstrate the same for managers. Second, we avoid the risk of measuring effects in the very short run that might dissipate after uncertainty is lifted. Third, two months is still short enough to largely rule out any confounding events that could have taken place between the first and second survey wave. Our findings indicate that anti-globalization sentiment has not spread, but that people have become more concerned about Austria’s foreign energy dependency. Based on our results, we would not expect an increase in general anti-globalization sentiment after the Russian invasion in other European countries. Yet, increased concerns about energy dependency were likely more widespread.

We contribute to a small literature, which exploits the invasion of Ukraine as a largely unexpected shock that may fundamentally change attitudes and expectations among experts or the public. Dräger et al. (2022) show that inflation expectations among German economists increased in the days after the invasion compared to before the invasion. The general public, however, needs more time to update its expectations (see also Ropele and Tagliabracci 2022). Steiner et al. (2023) study whether the invasion has affected attitudes toward European integration among exchange students from six European countries. One of their indicators also measures globalization attitudes. Steiner et al. study only a very short time period after the Russian invasion, during which they also find no change in general globalization attitudes. The Russia-Ukraine war of 2022 is not Russia’s first illegal invasion of Ukraine in recent years. Gehring (2022) shows that Russia’s annexation of Crimea in 2014 has increased trust in EU institutions and support for EU common policies, particularly in countries directly threatened by Russia’s territorial expansion.

Section 2 describes the data collection and presents some stylized facts. In Sect. 3, we proceed with the empirical analysis and interpretation of results, before Sect. 4 concludes.

## Data

Between February 14 and February 17, 2022, just before the Russian invasion of Ukraine, Gallup Austria conducted its regular sentiment barometer ("Gallup Stimmungsbarometer"), which included the first wave of the present survey. Fieldwork for the follow-up wave was carried out April 19 to April 22, after two months of war. The resulting economic sanctions were already in place, and threats of natural gas shortages and soaring food and energy prices present in the media. The sample size is 1000 respondents per survey wave, the results are representative of the internet-active Austrian population aged 16 and older. The interviews were conducted online (computer-assisted web interviewing) in the Gallup Online Access Panel.^2^

For structural conformity with the Austrian population aged 16 and older, stratified random sampling was used: In the first step, the distribution of socio-demographic characteristics (gender, age, federal province, occupation, education level, size of locality, households with children under 15 years of age) in the population was determined; in the second step, a purely random sample was drawn from the panel for each stratum. Deviations from the population in individual strata were subsequently corrected by weighting. Quotas were formed and weights calculated on the basis of the micro-census data from Statistics Austria. The samples only include internet users.^3^ A balance test reported in Table 7 in the “Appendix” shows that the socio-demographic characteristics of respondents in the two waves are identical.

Both survey waves asked respondents identical questions regarding (1) negative effects of economic globalization on Austria, and (2) whether Austria should become less dependent on imports. Respondents could express their conformity with the statement on a 4-point Likert-scale, ranging from completely agree to completely disagree. Answers are recoded such that higher values reflect stronger approval.^4^ In addition, both waves of the survey asked respondents, which topics on a list Austria needed to confront urgently and make them a priority. One topic on the list was (3) Austria’s independence from energy imports. The corresponding dummy variable is coded one if independence from energy imports is named as a political priority for Austria.^5^ Table 1 summarizes these three dependent variables before and after the Russian invasion and Table 2 provides a correlation matrix.

**Table 1:** Globalization attitudes in Austria before and after the Russian invasion

	Before (February) (%)	After (April) (%)
(1) “Economic globalization is bad for Austria.”	51.5	50.9
(2) “Austria should become less dependent on foreign imports.”	80.2	85.7
(3) “Priority issue: Independence from energy imports.”	45.8	58.1

(1) and (2): figures denote the share of respondents who completely agree or agree with the statement. (3): figures denote the share of respondents who consider this issue a policy priority

**Table 2:** Correlation matrix for globalization attitudes

	(1)	(2)	(3)
(1) Globalization	1		
(2) Import dependency	0.384***	1	
(3) Energy imports	− 0.021	0.188***	1

Pearson correlation coefficients, *p < 0.05; **p < 0.01; ***p < 0.001

Before the Russian invasion, the share of respondents who ‘agreed’ or ‘completely agreed’ that globalization is bad for Austria was 51.5%, and that share, if at all, decreased in the second survey wave (50.9%). In contrast, already before the invasion 80.2% (completely) agreed that Austria should become more independent from imports and this share increased even further to 85.7%. The most dramatic change in public opinion concerned whether independence from energy imports should be a political priority for Austria. The share of respondents who saw this as a priority increased from 45.8 to 58.1%.

## Empirical analysis

To study the impact of the Russian invasion of Ukraine on the attitude of Austrians towards globalization and Austria’s dependence on imports of energy or goods in general, we are estimating logit regression models. Dependent variables with an ordered categorical response are recoded such that (complete) agreement with a statement is one and (complete) disagreement is zero. This serves to make our results comparable across the dependent variables, since one of them is a binary indicator. However, all our results are robust to using the original indicators and estimating either ordered logit or linear regression models (i.e., linear probability models). All models are estimated using survey weights and robust standard errors. All robustness tests are reported in the Online Supplementary Information. Our independent variable of interest ($treatment$) is a dummy variable that indicates whether a survey response was provided before (0) or two months after (1) the invasion of Ukraine. We estimate the following estimation equation:

$$P(y = 1|x) = 1/(1 + e^{{ - (\beta_{0} + \beta_{1} x)}} ),$$

where $x$ is the vector of independent variables, including $treatment$ and socio-demographic respondent characteristics such as gender, age, education, or household income. To test for effect heterogeneity, we interact these respondent characteristics with our treatment variable. For ease of interpretation. these models are estimated as linear regression models, although again other estimators yield the same results.

The average marginal effects reported in Table 3 indicate, in line with the descriptive statistics in Table 1, that agreement with the statement that economic globalization is bad for Austria has not increased after Russia invaded Ukraine. This is in line with findings of Steiner et al. (2023) who study the attitudes of exchange students from six European countries before and after the invasion. At the same time, Austrians have become significantly more concerned about Austria’s dependency on imports. The estimated effect size of a 4–5-percentage-points increase is sizable.

**Table 3:** Globalization attitude changes in Austria after the Russian invasion

	*Globalization*		*Import dependency*		*Energy imports*	
	(1)	(2)	(3)	(4)	(5)	(6)
Treatment	− 0.012 (0.027)	− 0.017 (0.028)	0.050** (0.019)	0.041* (0.020)	0.117*** (0.024)	0.111*** (0.025)
Female		− 0.004 (0.030)		0.014 (0.020)		− 0.028 (0.026)
Age (vs. 16–30)						
Age 31–60		0.070 (0.041)		0.080* (0.031)		0.094** (0.036)
Age > 60		0.116** (0.044)		0.142*** (0.031)		0.246*** (0.040)
Education (vs. Low)						
Medium education		− 0.013 (0.040)		0.003 (0.025)		0.014 (0.035)
High education		− 0.119** (0.044)		− 0.104*** (0.031)		0.025 (0.040)
Income (vs. < 1501)						
1501–2500 EUR		− 0.013 (0.040)		0.033 (0.029)		0.021 (0.036)
2501–3500 EUR		− 0.045 (0.042)		0.018 (0.030)		0.032 (0.038)
> 3500 EUR		− 0.061 (0.042)		0.042 (0.029)		0.108** (0.038)
Observations	1621	1439	1876	1647	2000	1751
LR Chi^2^	0.19	31.54	7.13	71.27	23.37	71.76
Log likelihood	− 1123	− 966	− 841	− 712	− 1372	− 1142

Logistic regression models with survey weights, average marginal effect estimates reported with robust standard errors in parentheses, (1)–(2): economic globalization is bad for Austria, (3)–(4): Austria should reduce its dependence on imports, (5)–(6): priority for Austria: Energy, *p < 0.05; **p < 0.01; ***p < 0.001

For our control variables, we find that gender is not related to attitudes towards globalization and import dependency. Older individuals are more concerned about globalization and import dependency and more convinced that energy imports should be a political priority. Individuals with high education are less concerned about globalization and import dependency, but they are just as convinced as low-education individuals that energy imports should be a political priority for Austria. Household income does not seem to be important for globalization attitudes. Only members of high-income (i.e., over 3500€ per month) households appear to be more concerned about energy imports than others.

Next, we take a closer look at people’s perceived political priorities for Austria by comparing energy imports to possible alternative answers that could also be related to the invasion of Ukraine. Column (1) in Table 4 shows the results corresponding to Column (5) in Table 3. In Columns (2) to (7), the dependent variable is replaced by another dummy variable, reflecting a different political priority. Respondents are free to select multiple items as political priorities. The most obvious result of this empirical exercise is that no other policy issue than energy imports has increased in perceived importance in the two months after the invasion of Ukraine. This rules out that the increased concern about energy imports is part of a larger trend towards more concern about economic policies, which could have been either preexisting or a consequence of Russia’s invasion of Ukraine.

**Table 4:** Perceived priority changes for Austria after the Russian invasion

	(1)	(2)	(3)	(4)	(5)	(6)	(7)
Treatment	0.117*** (0.024)	− 0.057* (0.024)	0.011 (0.024)	− 0.076*** (0.023)	− 0.048* (0.022)	− 0.031 (0.023)	0.025 (0.022)
Observations	2000	2000	2000	2000	2000	2000	2000
LR Chi^2^	23.37	5.58	0.20	10.52	4.76	1.87	1.30
Log likelihood	− 1372	− 1361	− 1374	− 1293	− 1196	− 1277	− 1201

Logistic regression models with survey weights, average marginal effect estimates reported with robust standard errors in parentheses, Priority for Austria: (1): energy, (2): immigration, (3): environment, (4): unemployment, (5): public debt, (6): health and old age care, (7): inflation, *p < 0.05; **p < 0.01; ***p < 0.001

Three policy priorities have not changed at all due to the invasion (environmental and climate protection, old age and health care, and—interestingly—inflation). This indicates that although experts and citizens expected higher inflation rates following the invasion (see Dräger et al. 2022), this effect was not large enough by April to warrant an increased perception that inflation should be a policy priority for Austria. However, inflation has risen substantially in importance on the political agenda in the months following April 2022. Moreover, it should be noted that inflation was already a concern for about two thirds of the population at the beginning of 2022. This high starting value may also explain why the increase between February and April was moderate and not statistically significant. For three policy issues, we find a significant drop in perceived importance since the invasion of Ukraine. All three effects are significantly smaller than the increase in concern about energy security. Immigration has become less politically relevant, maybe because the admission of Ukrainian refugees has been less controversial than that of Syrians, Afghans, etc. in the years before. Unemployment was also perceived to be less politically important, most probably because of an unexpectedly good labor market development. From December 2021 to April 2022, official unemployment in Austria declined substantially from 336,276 to 254,755 persons. In general, other issues might have simply become less likely to be mentioned as a priority because they were crowded out by the perceived need to reduce Austria’s dependency on energy imports. This is consistent with the fact that no other issue has gained in importance.

Table 4 does not include categories for which we see no theoretical link to the invasion of Ukraine. These are crime, digitalization, culture, consumer rights, regionalism, gender equality, quality of media, pension reform, education reform, tax reform, and affordable housing. When estimating models for these dependent variables as a robustness test (results available on request), we find that only two policy priorities are significantly affected: Both, crime control and gender equality are less likely to be named as a policy priority after the Russian invasion of Ukraine.

To test whether our results are driven by particular groups of survey respondents, we interact our treatment variable with binary indicators for education levels, household income, political orientation, age, perceived financial stress, and perceived social status. Only one of these interaction terms is statistically significant (for results see Tables 5 and 6 in the “Appendix”), indicating a homogenous treatment effect across a variety of social groups. Individuals with high education levels, however, do not increase their support for the statement that Austria should become less dependent on imports.

## Conclusion

The Russian attack on Ukraine in February 2022 has caused severe disruptions of international trade and uncovered Austria’s economic dependency on energy imports. We use two waves of representative population surveys, conducted in Austria right before the invasion and two months after, to assess changes in the public’s attitude towards globalization and import dependency as a short-term reaction to the onset of war in Europe. Our results suggest that attitudes regarding globalization are differentiated: While anti-globalization sentiment in general has not spread further, people have become much more concerned about strategic external economic dependencies, especially in energy imports.

The fact that the outbreak of the war and subsequent economic turbulences raised public concerns about energy imports, but it did not substantially influence attitudes towards globalization in general, can be explained in different ways. For example, one may read it as a sign of an increasing public awareness of the complexity of economic linkages regarding energy production and consumption. The outbreak of the war uncovered risks of a lopsided dependency of imports from a single supplier. However, the idea of risk diversification through globalization has not yet spread sufficiently among the Austrian population to increase support for globalization in general.

Austria is an interesting case study, as it is highly dependent on gas imports from Russia and its population is generally skeptical of globalization. Thus, we read our results as an upper bound for the changes in attitudes in most other European countries caused by Russia’s invasion of Ukraine. Additional studies of changes in attitudes towards globalization in other European countries would be desirable but depend on the availability of comparable survey data shortly before and after the Russian invasion.

### Electronic supplementary material

Below is the link to the electronic supplementary material.

Supplementary file 1 (PDF 198 kb)

## Appendix

See Tables 5, 6 and 7.

**Table 5:** Heterogeneous treatments I, OLS

	*Import dependency*			*Energy imports*		
	(1)	(2)	(3)	(4)	(5)	(6)
Treatment (T)	0.259** (0.086)	0.113 (0.090)	− 0.094 (0.169)	0.108 (0.056)	0.099 (0.053)	0.162 (0.085)
Education (vs. Low)						
Medium education	0.161* (0.073)			0.028 (0.044)		
High education	− 0.078 (0.080)			0.072 (0.047)		
T*MedEdu	− 0.158 (0.101)			0.049 (0.066)		
T*HighEdu	− 0.259* (0.114)			− 0.041 (0.071)		
Income (vs. 0–1500)						
1501–2500 EUR		0.048 (0.082)			0.049 (0.048)	
2501–3500 EUR		− 0.129 (0.081)			0.044 (0.049)	
> 3500 EUR		0.014 (0.081)			0.097* (0.049)	
T*1501–2500 EUR		− 0.102 (0.122)			− 0.024 (0.071)	
T*2501–3500 EUR		0.123 (0.121)			0.023 (0.074)	
T* > 3500 EUR		− 0.069 (0.121)			0.055 (0.075)	
Ideology (vs. left-wing)						
Rather left-wing			0.074 (0.110)			0.082 (0.062)
Rather right-wing			0.246* (0.110)			0.035 (0.062)
Right-wing			0.226 (0.175)			− 0.015 (0.091)
T*Rather left-wing			0.244 (0.178)			− 0.026 (0.092)
T*Rather right-wing			0.160 (0.178)			− 0.062 (0.093)
T*Right-wing			0.370 (0.271)			− 0.147 (0.140)
Observations	1876	1647	1876	2000	1751	2000
R^2^	0.035	0.008	0.023	0.017	0.023	0.022

OLS coefficient estimates (survey weighted) with robust standard errors in parentheses, (1)–(3): Austria should reduce its dependence on imports, (4)–(6): priority for Austria: energy, constant omitted, **p* < 0.05; ***p* < 0.01; ****p* < 0.001

**Table 6:** Heterogeneous Treatments II, OLS

	*Import dependency*			*Energy imports*		
	(1)	(2)	(3)	(4)	(5)	(6)
Treatment (T)	0.112 (0.096)	0.297* (0.130)	0.042 (0.156)	0.077 (0.053)	0.228** (0.075)	0.052 (0.091)
Age (vs. 16–30)						
Age 31–60	0.335*** (0.070)			0.039 (0.041)		
Age > 60	0.308*** (0.074)			0.268*** (0.046)		
T*Age 31–60	− 0.033 (0.111)			0.099 (0.062)		
T*Age > 60	0.022 (0.114)			− 0.034 (0.069)		
Financial stress		0.072* (0.029)			− 0.019 (0.016)	
T*Financial stress		− 0.069 (0.045)			− 0.037 (0.025)	
Social status			− 0.080* (0.034)			0.004 (0.021)
T*Social status			0.019 (0.052)			0.025 (0.031)
Observations	1876	1863	1834	2000	1982	1941
R^2^	0.033	0.009	0.009	0.050	0.020	0.016

OLS coefficient estimates (survey weighted) with robust standard errors in parentheses, (1)–(3): Austria should reduce its dependence on imports, (4)–(6): priority for Austria: energy, constant omitted, **p* < 0.05; ***p* < 0.01; ****p* < 0.001

**Table 7:** Balance table

	Diff	*Control*		*Treatment*	
		N	Mean	N	Mean
Female	0.00	1000	0.51	1000	0.51
Age (vs. 16–30)		1000		1000	
Age 31–60	0.00		0.51		0.51
Age > 60	0.00		0.27		0.27
Education (vs. Low)		1000		1000	
Medium education	0.00		0.49		0.49
High education	− 0.01		0.33		0.32
Income (vs. < 1501)		881		870	
1501–2500 EUR	0.02		0.27		0.29
2501–3500 EUR	0.00		0.24		0.24
> 3500 EUR	− 0.01		0.24		0.24

^†^*p* < 0.10; **p* < 0.05

## Footnotes

1 The Freedom Party (FPÖ), a vocal right-wing populist and anti-globalization party in Austria, gained a vote share of 16.2 percent in the 2019 election of the national parliament, down from 26.0 percent in the 2017 ballot, as it was associated with tremendous political scandals. The party is moreover famous for its close political ties with Russia’s ruling party. Before the Russian invasion, FPÖ leaders frequently called for the lifting of supposedly self-damaging and pointless international sanctions against Russia. Cf. https://www.rferl.org/a/russia-austria-freedom-party-pact-putins-party/28185013.html. Despite some half-hearted distancing after the invasion, the FPÖ position regarding sanctions remained almost unchanged.

2 The Gallup Online Panel comprises more than 60,000 members and is operated in accordance with the latest international standard for market, opinion, and social research ISO 20252:2019, which requires compliance with various quality criteria. Data available on request from the authors.

3 In 2021, 91% of the Austrian population aged 14 and older were internet users (Integral 2022).

4 The exact wording of the two survey questions was (translated from German): “On a 1–4 points scale, how strongly do you agree with the following statement: (1) Economic globalization is bad for Austria. (2) Austria should become less dependent on foreign imports.” Respondents could also answer “don’t know”. These observations have been eliminated from our sample.

5 The exact wording of the survey question was (translated from German): “Which of these issues should we urgently address in Austria? Which topics have priority at the moment?”. The set of 18 possible answers included “Energy supply (independence from energy imports, etc.)”.
