# Supplementary material for: Has the Russian invasion of Ukraine reinforced anti-globalization sentiment in Austria?
Source: Empirica (Dordr). 2023 Mar 15;50(2):289–99. doi: 10.1007/s10663-023-09572-1 (PMC10014393; doi:10.1007/s10663-023-09572-1)
Supplement: Supplementary file 1 — (PDF 198 kb) [file 10663_2023_9572_MOESM1_ESM.pdf]

Supplementary Information

"Has the Russian Invasion of Ukraine Reinforced Anti-Globalization Sentiment in Austria?"

Empirica – Journal of European Economics

Jerg Gutmann (jerg.gutmann@uni-hamburg.de), Hans Pitlik, and Andrea Fronaschütz

1. Different versions of Table 3:

Table 3: Globalization Attitudes, OLS

	(1)	(2)	(3)	(4)	(5)	(6)
Treatment (T)	-0.010	-0.018	0.103**	0.096*	0.118***	0.112***
	(0.047)	(0.049)	(0.039)	(0.041)	(0.024)	(0.025)
Female		-0.007		0.047		-0.028
		(0.051)		(0.043)		(0.026)
Age 31-60		0.116		0.231***		0.093**
		(0.073)		(0.062)		(0.036)
Age 61+		0.224**		0.282***		0.245***
		(0.075)		(0.065)		(0.040)
Medium Education		-0.060		0.027		0.015
		(0.069)		(0.055)		(0.035)
High Education		-0.151*		-0.177**		0.025
		(0.076)		(0.063)		(0.040)
1501-2500 EUR		-0.087		0.015		0.021
		(0.072)		(0.059)		(0.036)
2501-3500 EUR		-0.159*		-0.048		0.032
		(0.073)		(0.060)		(0.038)
>3500 EUR		-0.188**		0.022		0.108**
		(0.069)		(0.060)		(0.039)
Observations	1,621	1,439	1,876	1,647	2,000	1,751
R^2	0.000	0.023	0.004	0.044	0.014	0.055

Note: OLS coefficient estimates (survey weighted) with robust standard errors in parentheses, (1)-(2): Economic globalization is bad for Austria, (3)-(4): Austria should reduce its dependence on imports, (5)-(6): Priority for Austria: Energy, constant omitted, *: p<0.05, **: p<0.01, ***: p<0.001.

Table 3: Globalization Attitudes, Ordered Logit

	(1)	(2)	(3)	(4)
Treatment (T)	-0.026	-0.050	0.262**	0.253*
	(0.101)	(0.106)	(0.095)	(0.102)
Female		-0.019		0.085
		(0.110)		(0.107)
Age 31-60		0.238		0.590***
		(0.160)		(0.153)
Age 61+		0.462**		0.629***
		(0.160)		(0.159)
Medium Education		-0.114		0.067
		(0.147)		(0.138)
High Education		-0.368*		-0.448**
		(0.165)		(0.156)
1501-2500 EUR		-0.163		0.011
		(0.155)		(0.150)
2501-3500 EUR		-0.321*		-0.165
		(0.158)		(0.151)
>3500 EUR		-0.383**		-0.002
		(0.148)		(0.153)
Observations	1,621	1,439	1,876	1,647
LR Chi^2	0.07	29.06	7.53	61.72
Log likelihood	-2020	-1758	-2065	-1761

Note: Ordered logit coefficient estimates (survey weighted) with robust standard errors in parentheses, (1)-(2): Economic globalization is bad for Austria, (3)-(4): Austria should reduce its dependence on imports, (5)-(6): Priority for Austria: Energy, constant omitted, *: p<0.05, **: p<0.01, ***: p<0.001.

Table 3: Globalization Attitudes, Logit

	(1)	(2)	(3)	(4)	(5)	(6)
Treatment (T)	-0.047	-0.069	0.364**	0.300*	0.475***	0.470***
	(0.108)	(0.115)	(0.136)	(0.145)	(0.098)	(0.108)
Female		-0.017		0.101		-0.119
		(0.121)		(0.146)		(0.110)
Age 31-60		0.285		0.492**		0.391*
		(0.167)		(0.184)		(0.153)
Age 61+		0.475**		1.011***		1.029***
		(0.181)		(0.213)		(0.172)
Medium Education		-0.053		0.027		0.060
		(0.161)		(0.209)		(0.147)
High Education		-0.484**		-0.697**		0.106
		(0.181)		(0.216)		(0.170)
1501-2500 EUR		-0.051		0.233		0.087
		(0.165)		(0.203)		(0.152)
2501-3500 EUR		-0.186		0.122		0.133
		(0.171)		(0.206)		(0.159)
>3500 EUR		-0.250		0.299		0.456**
		(0.170)		(0.209)		(0.163)
Observations	1,621	1,439	1,876	1,647	2,000	1,751
LR Chi^2	0.19	31.54	7.13	71.27	23.37	71.76
Log likelihood	-1123	-966	-841	-712	-1372	-1142

Note: Logit coefficient estimates (survey weighted) with robust standard errors in parentheses, (1)-(2): Economic globalization is bad for Austria, (3)-(4): Austria should reduce its dependence on imports, (5)-(6): Priority for Austria: Energy, constant omitted, *: $p<0.05$, **: $p<0.01$, ***: $p<0.001$.

Table 3: Globalization Attitudes, Logit (marginal effects)

	(1)	(2)	(3)	(4)	(5)	(6)
Treatment (T)	-0.012	-0.017	0.050^{**}	0.041^{*}	0.117^{***}	0.111^{***}
	(0.027)	(0.028)	(0.019)	(0.020)	(0.024)	(0.025)
Female		-0.004		0.014		-0.028
		(0.030)		(0.020)		(0.026)
Age 31-60		0.070		0.080^{*}		0.094^{**}
		(0.041)		(0.031)		(0.036)
Age 61+		0.116^{**}		0.142^{***}		0.246^{***}
		(0.044)		(0.031)		(0.040)
Medium Education		-0.013		0.003		0.014
		(0.040)		(0.025)		(0.035)
High Education		-0.119^{**}		-0.104^{***}		0.025
		(0.044)		(0.031)		(0.040)
1501-2500 EUR		-0.013		0.033		0.021
		(0.040)		(0.029)		(0.036)
2501-3500 EUR		-0.045		0.018		0.032
		(0.042)		(0.030)		(0.038)
>3500 EUR		-0.061		0.042		0.108^{**}
		(0.042)		(0.029)		(0.038)
Observations	1,621	1,439	1,876	1,647	2,000	1,751
LR Chi^2	0.19	31.54	7.13	71.27	23.37	71.76
Log likelihood	-1123	-966	-841	-712	-1372	-1142

Note: Logistic regression models with survey weights, average marginal effect estimates with robust standard errors in parentheses, (1)-(2): Economic globalization is bad for Austria, (3)-(4): Austria should reduce its dependence on imports, (5)-(6): Priority for Austria: Energy, *: $p<0.05$, **: $p<0.01$, ***: $p<0.001$.

2. Different versions of Table 4:

Table 4: Priority for Austria, OLS

	(1)	(2)	(3)	(4)	(5)	(6)	(7)
Treatment (T)	0.118***	-0.057*	0.011	-0.076**	-0.048*	-0.032	0.025
	(0.024)	(0.024)	(0.024)	(0.023)	(0.022)	(0.023)	(0.022)
Observations	2,000	2,000	2,000	2,000	2,000	2,000	2,000
R^2	0.014	0.003	0.000	0.006	0.003	0.001	0.001

Note: OLS coefficient estimates (survey weighted) with robust standard errors in parentheses, (1): Priority for Austria: Energy, (2): Immigration, (3): Environment, (4): Unemployment, (5): Public debt, (6): Health and old age care, (7): Inflation, constant omitted, *: p<0.05, **: p<0.01, ***: p<0.001.

Table 4: Priority for Austria, Logit

	(1)	(2)	(3)	(4)	(5)	(6)	(7)
Treatment (T)	0.475***	-0.233*	0.044	-0.334**	-0.235*	-0.141	0.124
	(0.098)	(0.099)	(0.098)	(0.103)	(0.108)	(0.103)	(0.109)
Observations	2,000	2,000	2,000	2,000	2,000	2,000	2,000
LR Chi^2	23.37	5.58	0.20	10.52	4.76	1.87	1.30
Log likelihood	-1372	-1361	-1374	-1293	-1196	-1277	-1201

Note: Logit coefficient estimates (survey weighted) with robust standard errors in parentheses, (1): Priority for Austria: Energy, (2): Immigration, (3): Environment, (4): Unemployment, (5): Public debt, (6): Health and old age care, (7): Inflation, constant omitted, *: p<0.05, **: p<0.01, ***: p<0.001.

Table 4: Priority for Austria, Logit (marginal effects)

	(1)	(2)	(3)	(4)	(5)	(6)	(7)
Treatment (T)	0.117***	-0.057*	0.011	-0.076***	-0.048*	-0.031	0.025
	(0.024)	(0.024)	(0.024)	(0.023)	(0.022)	(0.023)	(0.022)
Observations	2,000	2,000	2,000	2,000	2,000	2,000	2,000
LR Chi^2	23.37	5.58	0.20	10.52	4.76	1.87	1.30
Log likelihood	-1372	-1361	-1374	-1293	-1196	-1277	-1201

Note: Logistic regression models with survey weights, average marginal effect estimates with robust standard errors in parentheses, (1): Priority for Austria: Energy, (2): Immigration, (3): Environment, (4): Unemployment, (5): Public debt, (6): Health and old age care, (7): Inflation, *: p<0.05, **: p<0.01, ***: p<0.001.

3. Different versions of Table A1:

Table 5: Heterogeneous Treatments I, OLS

	(1)	(2)	(3)	(4)	(5)	(6)
Treatment (T)	0.259**	0.113	-0.094	0.108	0.099	0.162
	(0.086)	(0.090)	(0.169)	(0.056)	(0.053)	(0.085)
Medium Education	0.161*			0.028		
	(0.073)			(0.044)		
High Education	-0.078			0.072		
	(0.080)			(0.047)		
T*MedEdu	-0.158			0.049		
	(0.101)			(0.066)		
T*HighEdu	-0.259*			-0.041		
	(0.114)			(0.071)		
1501-2500 EUR		0.048			0.049	
		(0.082)			(0.048)	
2501-3500 EUR		-0.129			0.044	
		(0.081)			(0.049)	
>3500 EUR		0.014			0.097*	
		(0.081)			(0.049)	
T*1501-2500 EUR		-0.102			-0.024	
		(0.122)			(0.071)	
T*2501-3500 EUR		0.123			0.023	
		(0.121)			(0.074)	
T*>3500 EUR		-0.069			0.055	
		(0.121)			(0.075)	
Rather left-wing			0.074			0.082
			(0.110)			(0.062)
Rather right-wing			0.246*			0.035
			(0.110)			(0.062)
Right-wing			0.226			-0.015
			(0.175)			(0.091)
T*Rather left-wing			0.244			-0.026
			(0.178)			(0.092)
T*Rather right-wing			0.160			-0.062
			(0.178)			(0.093)
T*Right-wing			0.370			-0.147
			(0.271)			(0.140)
Observations	1876	1647	1876	2000	1751	2000
R^2	0.035	0.008	0.023	0.017	0.023	0.022

Note: OLS coefficient estimates (survey weighted) with robust standard errors in parentheses, (1)-(3): Austria should reduce its dependence on imports, (4)-(6): Priority for Austria: Energy, constant omitted, *: p<0.05, **: p<0.01, ***: p<0.001.

Table 5: Heterogeneous Treatments I, Ordered Logit

	(1)	(2)	(3)
Treatment (T)	0.619**	0.259	-0.203
	(0.210)	(0.228)	(0.382)
Medium Education	0.381*		
	(0.176)		
High Education	-0.214		
	(0.194)		
T*MedEdu	-0.333		
	(0.249)		
T*HighEdu	-0.616*		
	(0.276)		
1501-2500 EUR		0.103	
		(0.203)	
2501-3500 EUR		-0.376	
		(0.198)	
>3500 EUR		-0.019	
		(0.200)	
T*1501-2500 EUR		-0.232	
		(0.299)	
T*2501-3500 EUR		0.369	
		(0.297)	
T*>3500 EUR		-0.148	
		(0.302)	
Rather left-wing			0.090
			(0.253)
Rather right-wing			0.538*
			(0.254)
Right-wing			0.638
			(0.432)
T*Rather left-wing			0.579
			(0.406)
T*Rather right-wing			0.364
			(0.409)
T*Right-wing			1.138
			(0.672)
Observations	1876	1647	1876
LR Chi^2	54.09	15.72	35.04
Log likelihood	-2035	-1790	-2045

Note: Ordered logit coefficient estimates (survey weighted) with robust standard errors in parentheses, (1)-(3): Austria should reduce its dependence on imports, *: p<0.05, **: p<0.01, ***: p<0.001.

Table 5: Heterogeneous Treatments I, Logit

	(1)	(2)	(3)	(4)	(5)	(6)
Treatment (T)	1.140**	0.447	-0.294	0.435	0.400	0.655
	(0.372)	(0.304)	(0.431)	(0.227)	(0.213)	(0.349)
Medium Education	0.416			0.116		
	(0.244)			(0.181)		
High Education	-0.417			0.290		
	(0.240)			(0.193)		
T*MedEdu	-0.845*			0.197		
	(0.429)			(0.267)		
T*HighEdu	-0.992*			-0.168		
	(0.425)			(0.285)		
1501-2500 EUR		0.293			0.201	
		(0.251)			(0.195)	
2501-3500 EUR		-0.029			0.181	
		(0.244)			(0.201)	
>3500 EUR		0.353			0.391	
		(0.260)			(0.200)	
T*1501-2500 EUR		-0.299			-0.101	
		(0.410)			(0.288)	
T*2501-3500 EUR		0.173			0.092	
		(0.409)			(0.300)	
T*>3500 EUR		-0.421			0.238	
		(0.421)			(0.309)	
Rather left-wing			-0.064			0.331
			(0.325)			(0.255)
Rather right-wing			0.418			0.144
			(0.333)			(0.257)
Right-wing			0.048			-0.063
			(0.485)			(0.379)
T*Rather left-wing			0.849			-0.101
			(0.473)			(0.378)
T*Rather right-wing			0.557			-0.254
			(0.487)			(0.381)
T*Right-wing			0.943			-0.593
			(0.763)			(0.583)
Observations	1876	1647	1876	2000	1751	2000
LR Chi^2	51.49	9.33	21.40	28.92	28.94	35.96
Log likelihood	-814	-743	-833	-1368	-1171	-1364

Note: Logit coefficient estimates (survey weighted) with robust standard errors in parentheses, (1)-(3): Austria should reduce its dependence on imports, (4)-(6): Priority for Austria: Energy, constant omitted, *: $p<0.05$, **: $p<0.01$, ***: $p<0.001$.

4. Different versions of Table A2:

Table 6: Heterogeneous Treatments II, OLS

	(1)	(2)	(3)	(4)	(5)	(6)
Treatment (T)	0.112	0.297*	0.042	0.077	0.228**	0.052
	(0.096)	(0.130)	(0.156)	(0.053)	(0.075)	(0.091)
Age 31-60	0.335***			0.039		
	(0.070)			(0.041)		
Age 61+	0.308***			0.268***		
	(0.074)			(0.046)		
T*Age 31-60	-0.033			0.099		
	(0.111)			(0.062)		
T*Age 61+	0.022			-0.034		
	(0.114)			(0.069)		
Financial stress		0.072*			-0.019	
		(0.029)			(0.016)	
T*Financial stress		-0.069			-0.037	
		(0.045)			(0.025)	
Social status			-0.080*			0.004
			(0.034)			(0.021)
T*Social status			0.019			0.025
			(0.052)			(0.031)
Observations	1876	1863	1834	2000	1982	1941
R^2	0.033	0.009	0.009	0.050	0.020	0.016

Note: OLS coefficient estimates (survey weighted) with robust standard errors in parentheses, (1)-(3): Austria should reduce its dependence on imports, (4)-(6): Priority for Austria: Energy, constant omitted, *: p<0.05, **: p<0.01, ***: p<0.001.

Table 6: Heterogeneous Treatments II, Ordered Logit

	(1)	(2)	(3)
Treatment (T)	0.302	0.655*	0.272
	(0.230)	(0.309)	(0.374)
Age 31-60	0.838***		
	(0.171)		
Age 61+	0.702***		
	(0.178)		
T*Age 31-60	-0.093		
	(0.268)		
T*Age 61+	0.003		
	(0.277)		
Financial stress		0.215**	
		(0.071)	
T*Financial stress		-0.141	
		(0.108)	
Social status			-0.234**
			(0.084)
T*Social status			-0.005
			(0.124)
Observations	1876	1863	1834
LR Chi^2	48.47	18.42	21.41
Log likelihood	-2039	-2043	-2008

Note: Ordered logit coefficient estimates (survey weighted) with robust standard errors in parentheses, (1)-(3): Austria should reduce its dependence on imports, *: $p<0.05$, **: $p<0.01$, ***: $p<0.001$.

Table 6: Heterogeneous Treatments II, Logit

	(1)	(2)	(3)	(4)	(5)	(6)
Treatment (T)	0.212	0.741	0.322	0.320	0.934**	0.202
	(0.248)	(0.448)	(0.573)	(0.221)	(0.313)	(0.371)
Age 31-60	0.747***			0.167		
	(0.200)			(0.176)		
Age 61+	0.956***			1.097***		
	(0.238)			(0.199)		
T*Age 31-60	0.117			0.391		
	(0.318)			(0.260)		
T*Age 61+	0.523			-0.134		
	(0.386)			(0.295)		
Financial stress		0.071			-0.075	
		(0.088)			(0.065)	
T*Financial stress		-0.138			-0.155	
		(0.151)			(0.103)	
Social status			-0.129			0.016
			(0.120)			(0.084)
T*Social status			0.001			0.104
			(0.193)			(0.127)
Observations	1876	1863	1834	2000	1982	1941
LR Chi^2	52.28	7.56	7.59	85.02	31.80	26.11
Log likelihood	-815	-835	-822	-1335	-1352	-1329

Note: Logit coefficient estimates (survey weighted) with robust standard errors in parentheses, (1)-(3): Austria should reduce its dependence on imports, (4)-(6): Priority for Austria: Energy, *: $p<0.05$, **: $p<0.01$, ***: $p<0.001$.
